# Barriers to the employability of people with disabilities in the South African public service

**DOI:** 10.4102/ajod.v12i0.1178

**Published:** 2023-07-17

**Authors:** Warren P. Charles, Liiza Gie, Rhodrick N. Musakuro

**Affiliations:** 1 Department of Human Resource Management, Faculty of Business and Management Sciences, Cape Peninsula University of Technology, Cape Town, South Africa

**Keywords:** disability, barriers, employment, provincial government, labour market, public service

## Abstract

**Background:**

South African public sector efforts to employ people with disabilities (PWDs) in the post-apartheid have been less successful, resulting in a poor transformation record during the past 27 years (1994-2021) due to the failure to integrate PWDs into mainstream employment in government departments.

**Objective:**

The objective of this article is to identify and highlight some of the barriers to the employability of PWDs in the South African public service.

**Method:**

The research was framed as a case study within the transformative research paradigm. A typical department in the Western Cape provincial government was selected for the study. In-depth interviews were conducted with selected top managers within the Western Cape provincial government. Convenience sampling technique of the purposive sampling method was adopted to select targeted respondents (*n* = 10). Thematic analysis was employed to condense the data collected into a small number of significant themes. Atlas.ti version 7 was also used to enhance the analysis.

**Results:**

The study found, among others, that there are conceptual, infrastructural, managerial and organisational factors affecting the employability of PWDs in mainstream public service.

**Conclusion:**

The study concludes that barriers to the socio-economic progression of PWDs, including infrastructural and psychosocial factors, continue to prevail and cause the socio-economic marginalisation of PWDs.

**Contribution:**

The study contributes towards efforts aimed at the inclusion of PWDs in the workplace by offering both internal and society-wide actions. Internally, successful inclusion of PWDs involves eliminating barriers, removing bureaucracy, initiating management development for disability matters, introducing appropriate talent development measures, and implementing collaborative management of PWDs. External or society-wide measures include campaigns to demystify disability and change attitudes, engage society structures, and improve societal knowledge of disability.

## Introduction

It is believed that people with disabilities (PWDs) have not been successfully integrated within mainstream employment in government departments in South Africa (Ned & Lorenzo 2016), resulting in a poor transformation record over the past 27 years (1994–2021). Essentially, PWDs still face inhibiting barriers that exclude them from employment (Shakespeare et al. 2019; Vornholt et al. 2018). This study explored the barriers to the employability of PWDs in the South African public service. This study also took note of the efforts by the South African government to attain inclusiveness of PWDs in the labour market. As reported by Hart, Bohler-Muller and Hagg (2018), PWDs as a share of the South African workforce from 2002 to 2016 had never reached the 2% level. Earlier reports from the Public Service Commission (PSC) (2008) indicated a failure on the part of the public service to meet disability targets. Consequently, PWDs have remained in a vicious circle of exclusion and poverty over the years (Shakespeare et al. 2019:1). As such, there is a need for studies that explore the barriers to the employability of PWDs. There have been indications that employers lack the necessary strategies to effectively integrate PWDs within the workplace and there is a lack of supportive infrastructure for the integration of PWDs in public sector workplaces. In their study, Gida and Ortlepp (2007) found that a lack of accessible facilities and public transport prohibited South Africa’s top 100 organisations from absorbing PWDs in their workplaces. As the largest employer in South Africa, the South African public service led the need for accommodating PWDs in employment, by setting for itself a 2% target for disability equity. In the light of this, Oskouie et al. (2017) suggested that there has been a significant need for more theoretical work to expand context-specific knowledge on disability stigma and discrimination. Moreover, as observed in Hewko et al.’s (2018) study of stigma and discrimination, there is a greater need for studies that investigate the employability of PWDs in organisational settings as opposed to society contexts. Considering this, the purpose of this study was to identify and highlight barriers to the employability of PWDs in the South African public service. To achieve this, the research question for the study was: What are the barriers preventing the employment of PWDs in the South African public service and what factors seem to be contributing to the barriers?

## Literature review

A literature exploration of the notion of disability in various academic publications shows that there has been a shift in the conceptualisation of the meaning of disability. This shift has been linked to experts such as Oliver (1990) of the social model. The social model views disability as a social construct rather than a medical condition. The medical model, on the contrary regards disability as a disease or sickness (Sisti 2014). Although the social model and medical model provide preliminary and valuable insights into understanding the term ‘disability’, the World Health Organization (WHO) (2011) has recommended that there should be a balance in approach between the medical model and the social model when defining PWDs. Striking this balance is therefore essential in order to ensure that PWDs are provided with the necessary support and resources they need, thereby accommodating them more effectively in the workplace.

According to Sing (2012), there is no ‘country-specific’ definition of the term ‘disability’ in South Africa, and this has an implication on how PWDs can be identified in the workplace. Moreover, the lack of clarity may have serious implications for employers, as it limits their ability to provide the necessary support and resources to staff with disabilities, thereby limiting their potential to succeed. However, Sing (2012) goes on to cite a broad definition of disability approved by the South African government:

[*T*]he loss or elimination of opportunities to take part in the life of the community equitably with others that is encountered by persons having physical, sensory, psychological, developmental, learning, neurological or other impairments. (p. 164)

Even though this definition has various characterisations of disabilities, it does not consider the fact that many PWDs are not able to take part in the life of the community because of poor accessibility. This creates a fragmented understanding of disability and has the potential to result in a lack of consensus on what constitutes a disability and how it should be defined. Moreover, this in turn has implications for how disability is addressed in policy and practice.

**FIGURE 1:**
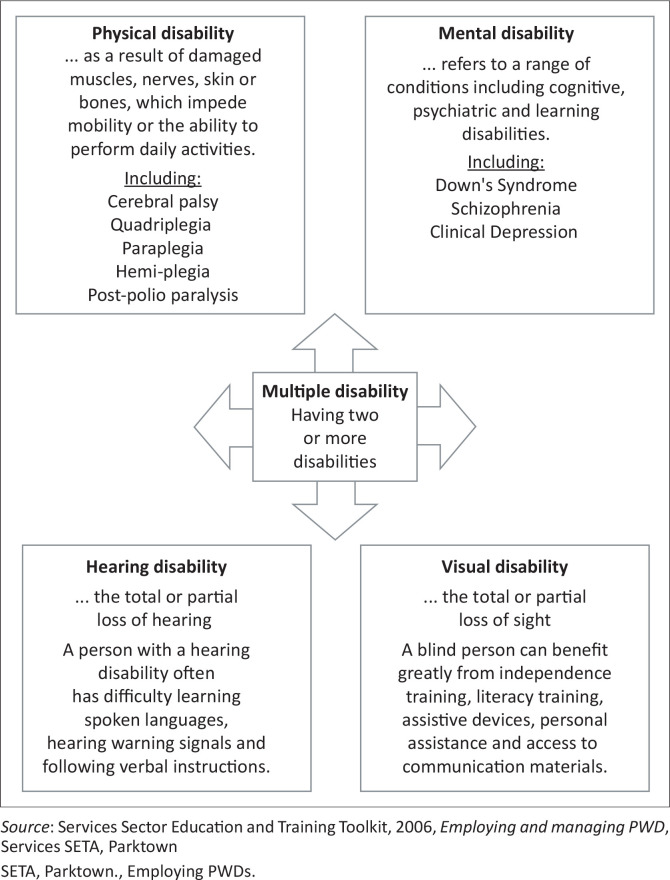
Categories of disability.

Considering that there are different models (social and medical) for conceptualising disability, the literature shows that one of the reasons why researchers have differing perspectives on the meaning of disability is that there are various categories and forms of disability. To this end, the Services Sector Education and Training Authority Toolkit (2006) provides categories of disabilities as shown in Figure 1. These categories are physical disability, mental disability, hearing disability and visual disability. Each category has its own unique set of challenges and needs that must be addressed in order to ensure that PWDs have access to the same opportunities in the workplace as those without disabilities. In the light of these categories and for the purposes of this study, disability is simply defined, in agreement with Cleaver and Unell (2011) as: ‘any form of physical, mental, hearing and visual disability condition that appears to impose limitations and significantly impairs an individual’s probability of entering into and advancing in employment’. This definition of disability is in line with the International Labour Organization’s (ILO) Convention on the Rights of Persons with Disabilities, which defines disability as a long-term physical, mental, intellectual or sensory impairment (Stein et al. 2007). Also, this definition is consistent with Grobler et al. (2006) who define disability as a physical or mental impairment that substantially limits life activities. Thus, disability is seen to have a direct negative impact on an individual’s ability to secure and build a successful career in the workplace.

One aspect that makes the conceptualisation of disability problematic is the difficulty of distinguishing between normal and abnormal situations characterising being able and being ‘not able’. For instance, it is not always possible to tell normal from abnormal mental conditions (Connellan 2021). Another challenge rests on the changing nature of conditions. As Bonaccio et al. (2020:1) argues, some disabilities and impairments are invisible, and employers often underestimate the number of PWDs who are qualified for a given position because of the intermittent nature of their impairments. This can lead to an underestimation of the number of PWDs who are actually capable of excelling in the position, as well as an undervaluing of the skills that those individuals can bring to the job. Even though these challenges exist, disability remains a reality everyone must deal with. This means that legislation must be flexible and adaptive; it should be able to accommodate changes in disability conditions (Sing 2012). It is also imperative that employers create an accessible and inclusive work environment for PWDs. Disability must be better understood and accepted by society, so that PWDs can live and work without stigma or discrimination (Oliver 1990).

After the 1994 democratic transition, the new government spelt out a need for transformation to ensure the participation of all in every dimension of human existence (Sing 2012). These calls had greater implications for all sectors for economic participation, reduction of poverty, social mobility and the advancement of employment equity in the labour market. According to WHO (2011), the participation of PWDs in the labour market tends to be lower than that of PWDs because of factors that affect both the supply side and the demand side of the labour market. The supply side of PWDs in the labour market considers the possession of skills, knowledge, competences and behavioural attitudes that are required in the labour market, whereas the demand side considers the availability of employers who are capable of absorbing PWDs in employment as well as the provision of a favourable environment for such employment. Developing countries such as South Africa often suffer from high unemployment and other labour absorption challenges that tend to favour the employment of some groups at the expense of others (Mitra 2010). This, in turn, can create a sense of inequality and unfairness among different groups of people in the labour market. Moreover, disability-based stigma and discrimination are global problems (Parker & Aggleton 2003). In other words, most social evils (such as poverty, unemployment, inequalities, violence, and abuse) are likely to have a detrimental effect on the staff with disabilities. In this way, it is evident that the people with disabilities are particularly vulnerable to the effects of social adversity. Furthermore, the paradigm shifts from medical theories to socially rooted conceptions of disability that focuses on the effective manipulation of social elements in order to manage, control and disentangle the complicating elements of disability have become prevalent.

The integration of PWDs into mainstream employment depends on removing or eliminating specific barriers in the labour market, some of which may be entirely perceptual. The social model has been significant in offering directions for a perceptual shift. Gida and Ortlepp (2007) conducted interviews to investigate the human resource implications of employing PWDs. In their study, challenges to the employment of PWDs were classified into three groups, namely environmental, attitudinal and resources. A significant number of respondents mentioned that the attitudinal barrier of the stigma surrounding PWDs is a crucial challenge to their employment. It should be noticed that attitudinal factors are often rooted in the model of disability. Other attitudinal factors included ignorance of issues relating to disability on the part of management. Another major challenge concerns finding suitable PWDs with the required skills (Gida & Ortlepp 2007). Both are contributors to the cycle of poverty and inequality affecting PWDs. The lack of adequate access to educational opportunities among PWDs manifests itself in their failure to secure suitable jobs. Efforts to disrupt this cycle must start with providing educational and training opportunities and be followed by interventions in the labour market. Another major challenge observed was the non-disclosure of disability by persons with disabilities themselves. This demonstrates their acceptance of societal labelling and adoption of an isolation strategy to deal with society’s negative perceptions. These tendencies reinforce the urgent need for societal reform to foster holistic improvement in the status of persons with disabilities and allow for their integration into mainstream employment.

Another important and similar compilation of the factors contributing to high unemployment rates among PWDs is provided in the White Paper on disability (South Africa White Paper 1997). The WHO (2011) observes that PWDs worldwide can undertake respected professions, engage in entrepreneurship, be self-employed or work in various responsibilities in both government and private enterprise. The paragraphs below present an analysis of significant barriers to the employment of PWDs. The formulation of any framework for the employment of PWDs must begin with a proper comprehension of the barriers to this and how they interact with other factors to affect the exclusion of PWDs. Inclusivity as a concept remains fundamental to South Africa as a democracy. The White Paper on disability (South Africa White Paper 1997) identifies the factors contributing to high unemployment among PWDs, which include: (1) low skills level, (2) inadequate education, (3) discrimination in workplaces, (4) unsupportive labour legislation, (5) poor enabling mechanisms, (6) inaccessible workplaces, (7) a lack of rehabilitation and training and (8) general high unemployment levels and inadequate access to information. The White Paper on disability (1997) notes that PWDs are often employed in the Departments of Welfare and Labour sheltered and/or protective workshops, private welfare organisations or by PWDs themselves. These jobs do not provide the PWDs with competitive economic power to support themselves and their families.

Disability-based discrimination and stigma are worldwide issues (Parker & Aggleton 2007). There has been a paradigm shift from medical to social conceptions of disability that focuses on effectively manipulating social elements to manage, control and disentangle the complicating elements of disability (Sisti 2014). Yet despite widespread campaigns, stigma and discrimination against PWDs remain strong and prevalent (Koodibetse 2015). Goffman is widely cited as the originator of stigma theory. Stigma is ‘a mark of disgrace associated with a particular circumstance, quality or person’ (Dos Santos et al. 2014). This definition mirrors that of Skinner and Mfecane (2004), who characterised stigma as ‘a deeply discrediting attribute that reduces an individual to someone who is in some way tainted and can therefore be denigrated’. A significant element of stigma is the ‘loss of social identity’ associated with it (Emlet 2007).

Stigma and discrimination are socially embedded markers of difference among people in particular social settings. Grinker (2021) observes that the term ‘stigma’ originated in ancient Greece, where the skin of criminals was marked so that they could be recognised and condemned by communities. The term has come to be associated with uncomfortable labelling and negative perceptions, like those associated with contracting human immunodeficiency virus (HIV) and/or aquired immunodeficiency syndrome (AIDS). Link and Phelan, cited in Grinker (2021), conceptualise stigma as ‘the interaction of labelling, stereotyping, separation, and discrimination by community members whose social, political, and/or economic power places them in a position perceived as superior’. Skinner and Mfecane (2004) suggest that ‘stigma and discrimination are political tools which the powerful use to protect their position by denigrating the weak and burdened groups’. Although this view holds significant appeal, it lacks an in-depth analysis of the trade-offs between power, politics, psychological variables and stigma.

Discrimination remains one of the significant barriers to the integration of PWDs in mainstream employment. The literature attributes discrimination against PWDs to negative attitudes and a lack of knowledge or awareness of the concept of disability (Maja et al. 2011), arguing that discrimination and its rationales are socially constructed phenomena (Gida & Ortlepp 2007). According to Maja et al. (2011), people without disabilities often see PWDs as inferior, demonstrating the necessity of adopting a social model in handling their employment.

The social model of disability holds that society disables and reifies disability, that is, if society did not take cognisance of disability, there would be no disability at all. Maja et al. (2011) argue that the physical environment, comprising equipment, infrastructure and machinery, limits the employment of PWDs. Appropriate physical infrastructure is required to enable the proper employment of PWDs. Morwane, Dada & Bornman (2021) conducted a literature review of barriers to the employment of PWDs, using the levels of barriers set out in the International Classification of Functioning, Disability and Health (ICF) framework. This framework envisages disability in terms of bodily functions and structure, limitations in performing activities, inability to participate, and context-related environmental or personal factors. This study highlighted the significant role that the environment plays in strengthening the notion of disability and the poor employability of PWDs. Similarly, Gida and Ortlepp (2007) found that physical environment elements such as parking facilities, transport facilities, and sitting and rest areas for PWDs were challenging. Maja et al.’s (2011) literature review on the employability of PWDs found widespread evidence that in industrial settings, many employers have failed to employ PWDs because of inaccessible buildings and infrastructure.

Therefore, the conduciveness of the physical environment significantly affects the employability of PWDs. Adapting to the environment could be considered costly or difficult by employers and requires commitment. For instance, technological developments within the Fourth Industrial Revolution (4IR) have made possible assistive devices and technologies capable of creating an enabling environment for the employment of PWDs. Mji and Edusei’s (2019) presentation at the Fifth African Network for Evidence-to-Action in Disability conference highlighted how African countries are trailing behind in adopting assistive technologies (ATs) like artificial intelligence and robotics that can improve the environment for the employment of PWDs. Convincing evidence exists of the critical role of ATs in enabling inclusive education. Mji and Edusei (2019) list some of the focal areas for adopting ATs to improve the integration of PWDs in mainstream employment, chief among them being government support in providing assistive devices and promulgating relevant legislation.

In South Africa, barriers to integrating PWDs into mainstream employment seem closely linked to developmental problems. Research suggests that stigma and discrimination remain significant obstacles to the employment of PWDs in African countries in comparison with nations of the developed world. Studies on the employment of PWDs have often highlighted the impact of stigma and discrimination. Stigma and discrimination often result in PWDs avoiding employment or deciding to leave their jobs. According to Oskouie et al. (2017), there is a significant need for more context-specific knowledge about disability stigma and discrimination, specifically in organisational settings as opposed to broader societal contexts. Studies that focus on stigma in the workplace are essential for increasing the inclusion of PWDs in mainstream employment. Marumoagae (2012) observes that employers face the challenge of ensuring that people with disability access the labour market in response to the South African government’s attempts to improve the levels and conditions of employment of PWDs.

Policy and legislation have been developed in South Africa to address challenges faced by PWDs in the labour force; however, the practical implementation is challenging. A study of Top 100 companies in South Africa by Gida and Ortlepp (2007) showed that the companies’ human resource management departments lacked a strategy for employing PWDs. Marumoagae (2012) seems to share this view, observing that not much has been done to deal with the actual problems that PWDs encounter in the labour market with a low absorption rate of PWDs in the labour market (Statistics South Africa 2014). South Africa has made strides at the national policy level but now faces an implementation problem. It is unclear whether the failure to implement policy is because of ignorance of policy or because these are not fit for the South African context. Additional research is required to determine the reasons for their slow implementation.

It is imperative to emphasise, however, that the foundations of the human rights approach can be found in the Republic of South Africa’s Constitution (Constitution of the Republic of South Africa 1996). This is further supported by international agreements such as the United Nations Convention on the Rights of Persons with Disabilities (United Nations 2006). This means that the Constitution of South Africa created a framework for protecting the rights of all people, regardless of their disability or background. It also sets a standard for other countries to aspire to in the protection of human rights, and these standards have been further strengthened by international agreements. This is consistent with a secular moral disability model, in which the inclusion of PWDs is based on the general acceptance that it is a good thing to do.

The South African public service was set up under the provisions of Section 197(1) of the Constitution of the Republic of South Africa (*Act 108 of 1996*) (South Africa. Constitution of the Republic of South Africa, 1996) (hereafter referred to as the constitution). The promotion of the rights of PWDs was an important aspect of service delivery assigned to the new public sector by the constitution. As argued by Fagin (2011:2), the post-1996 public sector had a new mandate to redress the shortcomings regarding justice, equality and inclusiveness associated with the apartheid government. Apartheid policies significantly prejudiced PWDs from the black population who lived in remote or marginalised areas (Morwane et al. 2021:2). Morwane et al. (2021:2) observe that this situation is not uncommon in African countries. In the South African case, the new public service was tasked with implementing wide-ranging changes to address the injustices of the apartheid era (South Africa. Department of Social Development 2016).

The Public Service comprises more than 1 million people, making it the largest employer in South Africa (Sing 2012:161). The public service falls under the administration of the Department of Public Service and Administration (DPSA) and is led by a responsible Minister. In its 2017/2018 Annual Report, DPSA stated that the public service of South Africa is made up of 44 national departments and 144 provincial departments. To promote effectiveness, accountability and achievement of quality service delivery, the South African Constitution established an independent and impartial PSC to report to Parliament about public service operations.

## Research method and design

The study was framed within the need to achieve social justice and inclusiveness for PWDs. The ontological assumptions of the transformative research paradigm underpinned the philosophical approach of the study. Mertens (2016) asserts that the transformative research paradigm seeks to advance human rights and attain mainstreaming of disadvantaged groups such as PWDs and women. This paradigm is thus concerned with transforming societies at both personal and community levels (Creswell & Creswell 2018). A fundamental concern for this study was to advance the emancipation of PWDs, who are a marginalised and disadvantaged group as defined by *South Africa’s Employment Equity Act 66*.

To address the barriers for the employability of PWDs from the perspective of the transformative paradigm, there was a need to explore in detail a particular case that allows understanding of contextual issues associated with any barriers observed. Consequently, a single case study research design was adopted to ensure in-depth appreciation of particular issues. The case study is considered to be an inquiry into a single event or a set of events of interest with an effort to understand it adequately (Zucker 2009). The single case study research design was chosen because of its potential to provide a detailed, in-depth understanding of a particular phenomenon in its natural context (Yin 2008). To be precise, a single case study design was chosen to allow for a deep exploration of the complexities of the case, the employment of PWDs in a single provincial government department in the Western Cape. As noticed by Creswell and Creswell (2018), case studies are unique in that they allow for detailed analysis cost effectively.

The case study as a qualitative approach produces contextually rich data for identifying and comprehending barriers to the PWDs’ employment in the South African public service (Christensen, Johnson & Turner 2015). Interviews as a key data collection strategy among qualitative studies were used. Face-to-face interviews were conducted to collect data as part of the qualitative research approach employed in this study. The main reason for employing face-to-face interviews for this study was to obtain comprehensive, in-depth and thorough data about a phenomenon (Hughes 2002).

In terms of data collection procedures, the researchers approached the Office of the Premier to explain the study and indicate the main questions to be answered during the interviews. The official suggested that the deputy directors and their assistants were the most appropriate respondents to be approached. At the end of the discussion, a memorandum of understanding (MOU) was signed and entered into with the provincial department. After signing the MOU, the researchers were taken on a tour of the department and introduced to staff members. The official from the Office of the Premier then sent out an email to all deputy directors and assistant directors, explaining the study and inviting them to participate. The full number of deputy directors and assistants in the department was not revealed, but 10 responded and indicated their willingness to participate in the study. To avoid work disruptions, the interviews were scheduled in a well-spaced manner over a 3-month period. The average time for an interview was 90 min.

Consistent with interviews, an in-depth interview guide that was largely open-ended was used to collect data. The open-ended interview guide was developed based on the research questions and the theoretical findings reached during the literature review. After the structured interview guide was designed, it was essential to ensure that it attended to the research objectives, it followed appropriate ethical guidelines and the questions were appropriate in terms of clarity, specificity, and relevance. To this end, a panel of experts in disability studies was assembled to scrutinise the interview guide.

Five experts made themselves available to ‘validate’ the interview guide. The experts had all published in various accredited journals and were known for their strength in conducting academic studies. The experts comprised three renowned academics, one member of the disability community and one senior employee in the South African public service. The experts sat three times to validate the research instrument at the beginning of the study. At the first sitting of the panel to discuss and validate the questions, some questions were eliminated, and others were proposed for inclusion. The panel of experts made recommendations for a review of the interview guide at the end of the first session. The research instrument was then amended to take into account the suggestions made. The instrument was then re-submitted for another review. The experts convened again and took note of the changes made. Minor corrections and suggestions were conveyed to the researcher via a report. After these matters had been attended to, the instrument was submitted for final review and pronounced ‘valid’, trustworthy and in line with the study’s problem.

To confirm that the interview instrument met the purposes of the study and could solicit the required data, an arrangement was made for it to be pretested. The researcher established contact with a provincial department other than the targeted one and requested an interview with a deputy director or assistant director. It was explained that the purpose was to ensure the instrument’s suitability for application to another provincial department. The deputy director who agreed to participate in this pretesting process was informed that the aim was to identify inappropriate or poorly constructed questions. The aims and objectives of the study were explained so that the bigger picture, perspective and orientation could be seen. This pretesting process confirmed that the research instrument was appropriate as all the items in the guide were satisfactorily understood and answered. After this process, the interview guide was considered appropriate, valid and ready for implementation.

The study population consisted of members of a selected Western Cape provincial government department. The department was selected according to the way the study was conceived; in that, it was considered a typical provincial government department concerning the integration of PWDs in mainstream employment.

The convenience sampling technique of the purposive sampling method was used to select respondents. Purposively, deputy directors and their assistants were selected to participate in this study because they’re the key employees responsible for the interpretation of the disability policy and the preparation of recruitment strategies at the Western Cape provincial government department. It was clear that, given its transformative orientation, the deputy directors and their assistants would meet the purpose of the study. Ritchie, Lewis and El Am (2003) note that transformative or critical studies employ ‘critical case’ sampling based on the purposive selection of participants who are likely to be essential to their purposes.

### Data analysis

Thematic analysis was used to condense the data collected into a small number of significant themes. Atlas.ti version 7 was also used to enhance the analysis by building network diagrams that showed important data patterns and linkages. After that, the study employed inductive themes, which are rich in context and more detailed, allowing discussions to be determined by data (Braun & Clarke 2006). The thematic process that was unfolded in this study respected the constant comparison techniques associated with grounded theory, during which researchers juxtapose emerging trends with ideas from previous studies (Glaser & Strauss 1967).

### Ethical considerations

Given the nature of the study, ethical considerations related to anonymity, confidentiality of data, informed consent and respect of participants were adhered to.

## Results

### Biographical details of respondents

Table 2 presents a demographic profile of the 10 respondents who took part in the study. Seven out of the 10 participants were deputy directors in the provincial government (PG) department selected for analysis in this study, while the remaining three were assistant directors. The respondents were, therefore, managers familiar with the strategic thrust of the department and fully aware of disability management issues within the department.

The average length of experience of the respondents as public service officers was 14 years, which underlines their seniority and capacity to offer meaningful and relevant information. With six of the respondents being female and four respondents being male, there was a reasonable gender balance among them. In summary, these characteristics rendered the respondents suitably qualified to provide in-depth data concerning the barriers to the employability of PWDs in the South African public service.

### Responses to interview question 2: What do you think are the barriers to integrate people with disabilities into employment within various departments?

Considering the second interview question, the data were first considered relative to the literature before it was matched with relevant codes deduced from the literature. These codes mainly emanated from the WHO (2011) guidelines on barriers to integrating PWDs into mainstream employment, which classified barriers as psychosocial and cultural, infrastructural, managerial and attributable to the nature of the organisation. The barriers identified in this study were extracted from the responses shown in Table 2.

The results shown in Table 1 were subjected to analysis facilitated by Atlas.ti to reveal relationships among themes, categories and sub-categories. Data display techniques are essential in analysis as they assist in depicting important links, patterns and relationships within the data (Verdinelli & Scagnoli 2013).

**TABLE 1:** Demographic profile of the respondents.

Respondent	Job title	Gender	Experience (years)
1	Deputy director	Female	6
2	Assistant director	Female	13
3	Assistant director	Male	3
4	Deputy director	Female	2
5	Assistant director	Male	11
6	Deputy director	Male	20
7	Deputy director	Female	30
8	Deputy director	Female	28
9	Deputy director	Female	12
10	Deputy directors	Male	14

**TABLE 2:** Thematic codes for interview question 2.

Respondent	Response summary	Theme	Category	Sub-category
1	Senior staff members are not knowledgeable on ‘Disabilities’ or type of disabilities. A barrier is that people have certain assumptions or opinions about people who are with disabilities. Resistance to change is another barrier, for example, when a person with disabilities is appointed, a lot of questions are being asked. Our workplace or infrastructure is not disability friendly. For example, within a schooling environment, children with learning abilities are being accommodated, but in the workplace, one still sees that a lack of reasonable accommodation.	Psychosocial and cultural barriers	The challenge of senior employees	A lack of knowledge resistance to change discrimination
		Infrastructural barriers	Infrastructure not disability friendly	Infrastructure and a lack of reasonable accommodation
2	There shouldn’t be any barriers, but unfortunately, there are. The first barrier is reasonable accommodation, hence we have a reasonable accommodation policy. So with the reasonable accommodation policy, the department should help or assist such a person to integrate. For example, if a person is appointed and the person is blind, there should be assistance given to the person with disabilities.	Managerial barriers	Reasonable accommodation	Workmate based assistance
3	Not aware of any barriers. The provincial government uses ‘Dial a ride’ where PWDs are integrated into the workplace by means of transport from their workplaces to work and back. So, transport for a person with disabilities might be a barrier, and ‘Dial a ride’ seems to address that specific need.	Technological infrastructure	Mobility assistive technology	Dial-a-ride
4	Barriers like not making the workplace wheelchair friendly. Another barrier can be hearing-impaired people not having someone to transcribe for them in meetings. The Department of Cultural Affairs has a special ramp built to address barriers for wheel-chaired individuals.	Infrastructural	Assistive devices and people	Wheelchair friendly infrastructure Transcribing Ramp for wheelchairs
5	The workplace might not be occupationally safe for people with disabilities. Conditions must be favourable for staff members with disability. There might be obvious barriers but I don’t see any barriers, but the department just needs to cater for people with special needs.	Nature of organisation	Safety	Special needs Work conditions
6	Staff members need a mind shift in terms of PWDs. For example, when you need to continually assist a disabled staff member, they might think it is a burden.	Psychosocial and cultural barriers	Attitudes	PWDs are a burden
7	There are a lot of stereotypes around abled staff towards disabled employees. Access to the building might be a barrier. These stereotypes can be a huge barrier within the workplace. Staff are not showing much empathy towards PWDs. Another barrier might be integration challenges; the building is not conducive for staff members with disability, although arrangements are made for disabled parking.	Psychosocial and cultural	Attitudes	Discrimination Stereotypes A lack of empathy
		Infrastructural	Assistive structures	Disabled parking Building structures
8	There are not a lot of barriers. A barrier can be a lack of awareness among staff members about types of disabilities. Issues around access and mobility when potential applicants with disabilities come for interviews.	Knowledge, psychosocial and cultural	A lack of awareness	-
		Infrastructural	Assistive devices	Devices to aid access Devices to assist mobility
9	A barrier might be the accessibility to the building, especially wheelchairs. A barrier can be proper planning, awareness and the necessary tools to assist staff members with disabilities.	Infrastructural challenges	Assistive devices	Wheelchairs Necessary active devices Access infrastructure
		Managerial challenges	Administrative	Proper planning Organising
10	A barrier might be the intent to appoint PWDs. Recruiting PWDs might be a problem. For example, we visit various municipalities and drive far distances. Driving engagements are onerous, and it might be that driving long distances might be a barrier to appointing PWDs.	Attitudes	Intent	Recruiting PWDS is a problem Mobility challenges

PWDs, people with disabilities.

**FIGURE 2:**
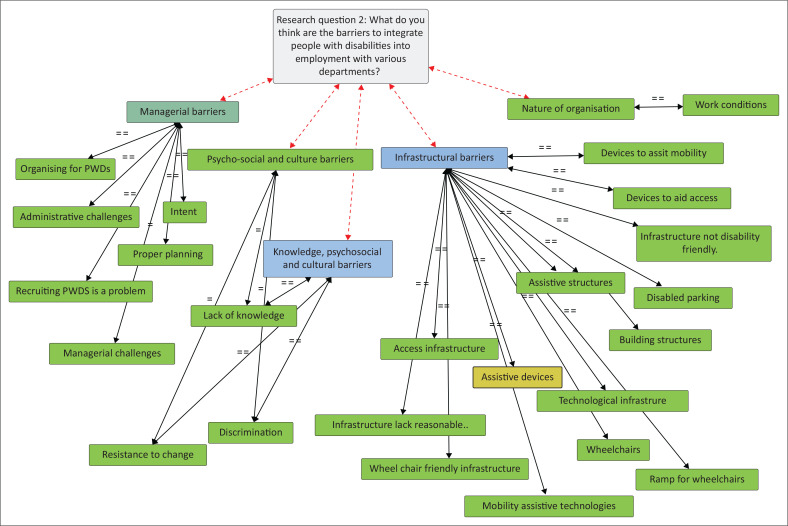
Barriers to the integration of people with disabilities.

As illustrated in Figure 2, this study found that respondents identified managerial, cultural, psychosocial, infrastructural and organisational barriers as critical variables affecting the possible integration of PWDs into mainstream employment within the provincial government. In the case of managerial barriers, the findings support those of Bonaccio et al. (2020) that managers often found themselves in a dilemma as a result of the ineffectuality in the performance of their key management tasks regarding PWDs. Some respondents were also of the view that there was a ‘lack of will’ to achieve goals relating to the integration of PWDs in mainstream employment. Respondents also mentioned that it was difficult to simply accept working with PWDs, thereby exhibiting negative attitudes towards the employment of PWDs. This seems to account for the low absorption of PWDs into mainstream employment.

### Responses to interview question 3: What challenges do people with disabilities face in finding employment within the Western Cape provincial government?

Inductive thematic analysis of the challenges faced by PWDs in finding employment within the PG department generated themes that were earlier established under the heading of barriers to the employment of PWDS (Table 3). In other words, organisational, conceptual, integration approach and infrastructural challenges emerged from the thematic analysis, as shown in Table 4. There were, however, some respondents who felt that PWDs did not face any challenges in acquiring employment within the PG department. Their views seemed to resonate with the study’s findings of previous studies that reported that there is a degree of ignorance of problems and issues affecting PWDs in the labour market (Gida & Ortlepp 2007).

**TABLE 3:** Barriers to the employment of people with disabilities.

Nature of the challenges	Specific aspects	*N*
Environmental	Inaccessible facilities	3
	Nature of the industry	3
	Inaccessible public transport	1
Attitudinal	Stigma attached to people with disabilities	8
	Ignorance of issues related to disabilities	6
	Not enough will power and Involvement from top management	4
	Lack of understanding from colleagues and managers	1
	Management’s view that it is just one of HR’s interventions	1
	Disabilrty still perceived as a social responsibility issue	1
Resources	Difficulty in finding PWDs who have the right skills for the jobs available	8
	Costs of accommodation required for various types of disabilities	6
	Non-disclosure of disability by employees who have disabilities	4
	Provision of medical aid cover	1
	Not enough role models to learn from	1

*Source*: Gida, P. & Ortlepp, K., 2007, ‘Employment of people with disabilities: Implications for human resource management practices’, *Acta Commercii* 7(1), 135–150. https://doi.org/10.4102/ac.v7i1.21

PWDs, people with disabilities.

**TABLE 4:** Thematic template for interview question 3.

Respondent	What challenges do PWDs face in finding employment within the provincial department	Theme	Category	Sub-category
1	Departments are not ready and prepared to accept PWDs within the workplace. Hence, many departments are not ready to accommodate them. For example, there are no brail policies or communication for blind workers, and certain departments are not *wheelchair friendly*. I would think these are the things applicants with disabilities look at the environment where they might work in.	Organisational	Readiness	Managerial infrastructural.
2	The provincial government has a 2% target to appoint PWDs, not sure if the targets are being met or even the target is too low. The 2% criteria are there to accelerate the appointment of PWDs.	Organisational	Managerial	Strategic planning evaluation.
3	I don’t foresee any challenges. Sometimes, there are n**o** clear definitions in terms of what a disability entails. I was wheelchair-bound for 6 months because of an accident and the workplace was made accessible and accommodative for my mobility challenges. I don’t foresee that PWDs might have challenges finding employment within WCPG. Job vacancies especially imply that PWDs can apply, so the department attempt to eradicate any challenges for PWDEs.	Conceptual	Clarity	Definition of disability.
4	I don’t foresee any challenges that PWDs might encounter. I haven’t seen one application for our unit or department. I personally think that they are maybe are not applying. It might be that the directorate when advertising post, not using the correct platform attracting PWDs. For example, competent blind people might not be able to read a newspaper or computer.	Integration approaches	Inappropriate integration approaches	Wrong job advertisement platforms, a lack of job applicants from PWDs.
5	I don’t foresee any challenges that PWDs might encounter. PWD applicants do get preferential treatment to accommodate them.	No challenges	Preferential treatment	-
6	I don’t foresee any challenges.	No challenges		-
7	The Employment Equity header on job advertisements is sometimes not visible. Not all municipalities are conducive or accessible for PWDs. Usually, most positions held by PWDs are office bound, but our unit ‘Property rights’ is not office based. We do quarterly workshops in the Worcester training centre, but to integrate outside PWDs, the workplace is not accessible. In our unit, travelling is a priority; hence, I think PWDs might be reluctant to apply.	Organisational challenges	Infrastructural	Inaccessible organisations, employment equity not clear.
8	The issue of transport to come to an interview surely might be a challenge for a potential PWD job applicant, access to job interview, etc.	Organisational	Infrastructural	Accessibility.
9	I think there might be challenges in relation to access to information, accessibility and transport to interviews. The department might not use disability-friendly platforms to advertise post.	Infrastructural	Accessibility	Inaccessible workplaces, Limited access to information, no transport.
10	I don’t foresee any challenges, as the recruitment portals are open to all. A challenge might be mobility.	No challenges	-	-

PWDs, people with disabilities; WCPG, Western Cape provincial government.

**FIGURE 3:**
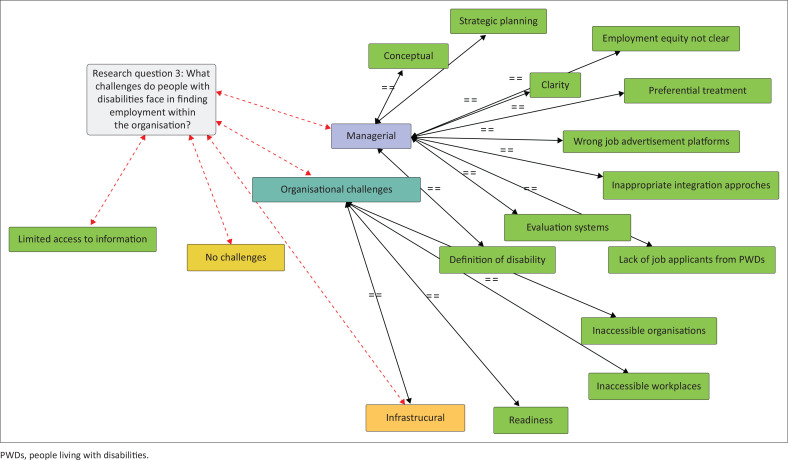
Network diagram of responses to the challenges faced by people with disabilities in finding employment within the organisation.

The responses provided in Table 4 were then configured on Atlas.ti to review relationships and categories that addressed the research question. The network diagram thus generated is presented in Figure 3.

As portrayed in Figure 3, there are managerial, organisational and conceptual challenges inhibiting the integration of PWDs into mainstream employment. In addition, some respondents expressed the view that limited access to information remains another barrier faced by PWDs. Some respondents felt that PWDs did not face any challenges. These findings are further discussed below.

### Access to information

This can be linked to managerial inadequacies. Respondents highlighted the use of the wrong job advertisement platforms that were inaccessible to PWDs or posed challenges to their effective recruitment. The main job advertisements used by government departments are newspapers and their own internet websites. A study by Maja et al. (2011) reveal that PWDs face significant marginalisation in the education system and therefore lack the skills and experience to compete in the mainstream job recruitment market. They may therefore require other recruitment methods and platforms.

### Absence of challenges

As observed in earlier discussions, some respondents seemed completely oblivious or ignorant of the challenges faced by PWDs. They argued that PWDs receive preferential treatment, are supported by affirmative action initiatives and do not face any challenges in recruitment. Some respondents believed that the challenges they faced were no different from those faced by able-bodied persons and were therefore unable to pinpoint anything specific.

### Responses to interview question 5: What in your opinion is the main reason why people with disabilities might find employment difficult within the WCPG?

Question 5 was a factual question and yielded the factual responses set out in Table 5. Opinions on the barriers to the employment of PWDs were wide-ranging but significantly resembled those discussed in the literature review chapters. Some respondents also felt that the attitudes, thoughts and inferiority complexes inherent among PWDs prevent them from getting absorbed into mainstream employment. Reasons for PWDs’ failure to find employment within the provincial government department can be identified from the perspective of the PWDs themselves as well as from the perspective of other people within the PG. As suggested by respondent 5, PWDs feel inferior and lack the confidence to join mainstream employment, while employees hold negative perceptions of the PWDs already employed. This is shown in Table 5.

**TABLE 5:** The main reasons for the failure to integrate people with disabilities into mainstream employment within the PG department.

Respondent	Reasons why PWDs might find it difficult to be employed at the PG department
1	Current workforce is not knowledgeable on the topic PWDs. Workers with disabilities don’t find acceptance within the workplace, but rather pity from co-workers. Staff need to get a level of understanding, acceptance and change-management with PWDs.
2	Difficulty in accessing employment opportunities as the portals that they use to attract PWDs are not accessible for PWDs. The mainstream advertising media might not even be PWD friendly.
3	I don’t foresee any difficulties for PWDs finding employment within WCPG.
4	Logistics getting to interviews or meetings. Locations can be a barrier.
5	The PWDs might have wrong assumptions while applying or viewing advertised positions. Feeling inferior when applying, even while the positions might be earmarked for a person with disabilities. There might be fears of the unknown or being accepted within the environment.
6	Many PWDs might not feel welcome. A lack of necessary support available to them. Staff might even see them as a burden.
7	Parking challenges for them. Buildings and toilets are PWD friendly. Mostly the fear of the unknown.
8	Wrong assumptions and mindsets from PWD applicants. There actually shouldn’t be any reasons.
9	Advertisement of job opportunities not communicated on PWDs’ career portals, hence a lack of access to engage opportunities. Staff might not also be sensitive in engaging PWDs.
10	Building in the department does not cater for staff with disabilities. Access to the building might be difficult. Load shedding, if you are an applicant in a wheelchair on the 9th floor and the lifts are not working that already impedes job applicants.

PWDs, people with disabilities; PG, provincial government; WCPG, Western Cape provincial government.

The other main reasons provided in Table 5 can be seen within the psychosocial, infrastructural and organisational framework established for earlier questions. The responses, however, provide another dimension that requires consideration: the attitudes of PWDs themselves, who tend to suffer from certain complexes that prevent them from taking up challenges that characterise the labour market. Respondent 10 also raised the issue of electricity load shedding in South Africa as potentially impeding the mobility of PWDs. This is because electricity load shedding can cause elevators and other disability-related equipment to stop working. This in turn can lead to mobility limitations for PWDs, as they are unable to access certain areas that require the use of elevators. There were also responses relating to the lack of knowledge about disability among employees already employed in the organisation. This lack of knowledge about disability conceptually limits the ease with which PWDs can be accepted in the workplace.

## Discussion

The lack of integration of PWDs into mainstream employment was found to be the result of conceptual, infrastructural, managerial and organisational issues. The themes isolated in the data analysis demonstrated that there were strong organisational and managerial challenges. This endorses the findings of Sing (2012), who argues that government departments have not succeeded in fully accommodating PWDs in their employment and structural systems. When this is considered in the light of the marginalisation and exclusion of PWDs during the apartheid era, it can be concluded that there has been deep-rooted failure in the transformation of South African society (Mahlangu 2009).

In the earlier literature review, it was shown that apartheid systems were characterised by the exclusion of certain categories of people from mainstream participation in socio-economic activities. The apartheid administration emphasised the principle of the separation of races, which significantly affected PWDs from the black population living in remote or marginalised areas (Morwane et al. 2021). In concert with the findings of previous research, this study has identified several barriers inhibiting the integration of PWDs, including the absence of a broad collaborative strategy for disability management and a centralised approach to disability management.

The failure to address the situation of PWDs seems to point to profound structural inadequacies in the public service (Sing 2012). These structural dimensions include a hierarchical structure in government institutions and a reliance on bureaucracy and directive-based management. The study found that issues concerning PWDs are sensitive and evoke a variety of emotions and attitudes. Such issues nevertheless need to be addressed to remediate past failures and promote the transformation agenda in South Africa.

It appears that while there has been some progress in the implementation of employment equity, especially in terms of the accommodation of all racial groups in mainstream employment, PWDs seem to be lagging behind. Further research into the extent to which compounded marginalisation is still prevalent in South African society may be necessary, for instance, by investigating how the situation of PWDs relates to factors of gender, race and economic status.

The discoveries articulated in this study include: (1) as a result of the failure to integrate PWDs into mainstream employment, they continue to suffer from socio-economic marginalisation despite the end of apartheid; (2) because of the failure to integrate PWDs into mainstream employment, the discrimination and segregation of PWDs have prevailed relative to other previously disadvantaged groups; and (3) because of the failure to integrate PWDs into mainstream employment, the present integration strategy must be ineffectual.

The public service is expected to take the lead in implementing government programmes and initiatives. Its failure to address an issue of national concern such as this means that the issue remains problematic in the private sector. If government departments can take the lead by employing the right strategy, private-sector employers are highly likely to follow suit.

Reasons such as lack of access, lack of information, discrimination and general lack of trust in being accommodated in the workplace have been identified as factors that continue to reduce the participation of PWDs in mainstream employment. Reasons for the failure to integrate PWDs into mainstream employment were found to be both internal and external and mutually reinforcing. Internally, there are psychosocial cultural barriers, infrastructural barriers and barriers inherent in the very nature of the organisation. External or societal factors included negative attitudes and discrimination, which were simply transferred to the workplace. The prevailing context of socio-economic marginalisation was thus found to have a major impact on workplace practices. The continued socio-economic marginalisation of PWDs in the community at large results in their failure to access basic amenities such as education and health. This further worsens their situation and means that they may lack the requisite qualifications and skills to be accepted into mainstream employment (Fagin 2011).

As PWDs cannot access other essential services such as healthcare, they are in dire need of infrastructural and personal support, which further accentuates their marginalisation and makes obtaining employment doubly difficult. Even if they gain employment, they remain strongly discriminated against and face serious limitations in progressing up the employment ladder. The result is a vicious circle of discrimination and marginalisation that needs to be addressed in both internal and external systems if the transformation agenda is to be advanced.

### Managerial implications

It is imperative that the South African public service addresses managerial barriers. The challenges related to mainstreaming PWDs into public service employees are influenced by the actions of leaders. Leadership development initiatives may be needed to enhance the capacity of public managers and administrators to ensure the successful integration of PWDs.

Accommodation infrastructure should be considered to promote workplace access for PWDs. In particular, the use of robots can be essential to improving the integration of PWDs. However, to ensure the effective adoption of disability friendly infrastructure, a disability champion should be appointed from within the organisation to carry out assessments of workplace accessibility. Such a disability champion can oversee all matters concerning disability.

The South African public service should also foster a disability friendly organisational culture. A disability friendly organisational climate will address psychosocial issues such as discrimination and stigma that affect PWDs in the workplace. It has been recognised that disability issues are largely shaped by the social environment. This includes: (1) culture, (2) social inequalities, (3) status, (4) political and power dynamics, (5) poverty, (6) abuse, (7) violence, (8) religion and more.

It is also imperative that the South African public service consider adopting society-wide strategies to improve the employability of PWDs. The recommendation is derived from the observation that public institutions are scrutinised by the general public and are affected by society’s events. The adoption of society-wide strategies for PWDs, therefore, appears critical. This study has established the need to address the psychosocial and cultural impediments to facilitating employment initiatives for PWDs. Once public attitudes start to shift, it will become much easier to handle disability in the workplace. Society-wide strategies should include dialogue with partners such as non-profit organisations, community leaders, churches, political institutions, non-governmental organisations and other community-based groups who are mostly concerned about the welfare of PWDs in the communities in which the public service sector operates.

The South African public service should also consider increasing disability-based advocacy. Advocacy helps to educate employees in the workplace including those in charge of talent management practices as well as members of the community on disability matters. This is performed by advancing the interests of PWDs in general. In other words, the successful integration of PWDs into mainstream employment will be expedited by vigorous advocacy to eliminate socio-cultural and attitudinal barriers to disability.

### Limitations and suggestions for future research

This study adopted qualitative research approaches that involve exploring the viewpoints and experiences of participants. The kind of data collected and the data collection methods could be changed in favour of other methodologies to offer different perspectives. For instance, a quantitative study based on structural modelling would complement and increase the authority of this one.

Moreover, this research comprised a single case study of one provincial government department. Future studies might look more widely at the public service in South Africa or compare public service with private sector attempts to accommodate PWDs.

## Conclusion

This study sought to identify and understand barriers to the employability of PWDs in the South African public service. Barriers to the socio-economic progression of PWDs, including infrastructural and psychosocial factors, continue to prevail and cause the socio-economic marginalisation of PWDs. The lack of integration of PWDs into the mainstream was found to be because of conceptual, infrastructural, managerial and organisational themes that complicated the integration of PWDs into mainstream employment. The themes demonstrated that there were strong organisational and managerial challenges that government departments still have a poor record in respect of the accommodation of PWDs in their employment and structural systems. When this is considered in relation to the marginalisation and exclusion of PWDs that was experienced in the apartheid era administration, it can be concluded that these findings point to deep-rooted failures in the transformation of South African societies as also argued in related literature on disability, societal transformation and development. The results of this study contribute to attempts to increasing the state of labour market equalisation in South Africa.
